# Current perspectives on the hormonal control of seed development in Arabidopsis and maize: a focus on auxin

**DOI:** 10.3389/fpls.2014.00412

**Published:** 2014-08-25

**Authors:** Antonella Locascio, Irma Roig-Villanova, Jamila Bernardi, Serena Varotto

**Affiliations:** ^1^Department of Agronomy Food Natural Resources Animals Environment - University of PadovaPadova, Italy; ^2^IBMCP-CSIC, Universidad Politécnica de ValenciaValencia, Spain; ^3^Dipartimento di Bioscienze, Università degli Studi di MilanoMilan, Italy; ^4^Istituto di Agronomia Genetica e Coltivazioni Erbacee, Università Cattolica del Sacro CuorePiacenza, Italy

**Keywords:** seed development, maize, Arabidopsis, endosperm, embryo, phytohormones, auxin

## Abstract

The seed represents the unit of reproduction of flowering plants, capable of developing into another plant, and to ensure the survival of the species under unfavorable environmental conditions. It is composed of three compartments: seed coat, endosperm and embryo. Proper seed development depends on the coordination of the processes that lead to seed compartments differentiation, development and maturation. The coordination of these processes is based on the constant transmission/perception of signals by the three compartments. Phytohormones constitute one of these signals; gradients of hormones are generated in the different seed compartments, and their ratios comprise the signals that induce/inhibit particular processes in seed development. Among the hormones, auxin seems to exert a central role, as it is the only one in maintaining high levels of accumulation from fertilization to seed maturation. The gradient of auxin generated by its PIN carriers affects several processes of seed development, including pattern formation, cell division and expansion. Despite the high degree of conservation in the regulatory mechanisms that lead to seed development within the Spermatophytes, remarkable differences exist during seed maturation between Monocots and Eudicots species. For instance, in Monocots the endosperm persists until maturation, and constitutes an important compartment for nutrients storage, while in Eudicots it is reduced to a single cell layer, as the expanding embryo gradually replaces it during the maturation. This review provides an overview of the current knowledge on hormonal control of seed development, by considering the data available in two model plants: *Arabidopsis thaliana*, for Eudicots and *Zea mays* L., for Monocot*s*. We will emphasize the control exerted by auxin on the correct progress of seed development comparing, when possible, the two species.

## Introduction

In order to ensure their continuation, Spermatophytes (Gymnosperm and Angiosperm plants) adapted seed development, a product of their sexual reproduction, which permits the maintenance of their lineages, allows them to be spread in the environment, and when needed, provides resistance during unfavorable environmental conditions (through the state of dormancy).

The seed comprises three compartments: embryo, endosperm and seed coat. The embryo represents the structure of the future adult plant. It encloses all the elements and fundamental patterns necessary for the new plant to develop after germination. The endosperm constitutes the reservoir for all the nutrients that the embryo will use during development and until the new plant becomes autotrophic. The seed coat derives from the integuments of the ovule and protects the vital part of the seed from mechanical injury, predators and drying out.

The seed originates from a double fertilization event, in which one sperm cell fertilizes the egg cell of the megagametophyte generating the diploid embryo, and a second sperm cell fertilizes the diploid central cell, from which derives the triploid endosperm (Reiser and Fischer, 1993; West and Harada, 1993; Goldberg et al., 1994).

**Figure 1:**
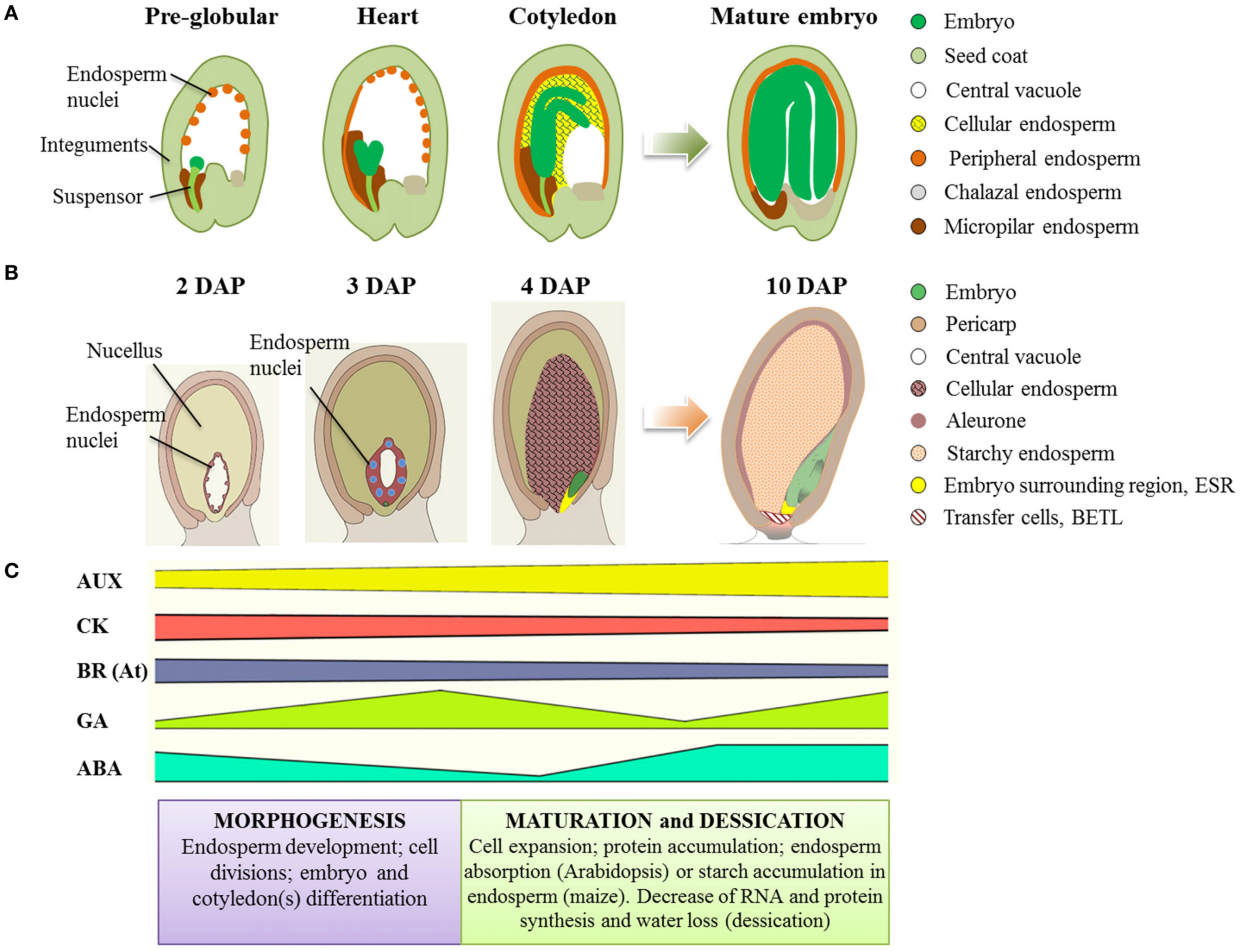
**Seed development in Arabidopsis and maize. (A)** Schematic representation of seed development in Arabidopsis. Embryo development stages are indicated. The evolution of the endosperm is shown from the formation of the coenocyte, where the multiple anticlinal cell divisions generate nuclei placed all around the peripheral cytoplasm, followed by the formation of the peripheral endosperm layer. This layer evolves into the cellular endosperm after periclinal divisions and cell wall formation events. Later in the developmental program, the volume of the central vacuole progressively decreases to finally disappear, and the endosperm is absorbed almost completely and replaced by the growing embryo in the mature seed. At the end of maturation only three types of endosperm remain: the single-cell layered endosperm, the micropylar endosperm surrounding the embryo radicle, and the chalazal endosperm, adjacent to the chalazal cyst. **(B)** Schematic representation of seed development in maize. Stages indicate days after pollination (DAP). In parallel with Arabidopsis, the progression of seed development is showed from the definition of the coenocyte, to the cellularization of the endosperm and progressive disappearance of the central vacuole. The process of maturation, besides others modifications, ends with the expansion of the endosperm that finally occupies the largest part of the seed and the accumulation of starch in its cells that progressively undergo programmed cell death. **(C)** Schematic trend of hormone accumulation during seed development. The high level of auxin (AUX) present during all the seed development phases suggests that this hormone has a key role throughout the entire program of seed formation. The pattern of Cytokinins (CK) accumulation is the opposite with respect to auxin. CKs have a prominent role during the phase that involves cell divisions, decreasing progressively during the maturation phase, when cell expansion prevails. The brassinosteroids (BR) follow the same pattern of CKs. The highest concentration of BRs is shown at the beginning of seed development, and is detected in the maternally derived tissues (i.e., integuments). Their levels decrease at the end of maturation. The pattern of accumulation of Gibberellins (GA) is characteristic, showing two peaks corresponding to specific phases of seed development: the stage of embryo differentiation, when the GAs promote cell growth and expansion, and the end of the maturation phase, when they activate proteolytic enzymes that mobilize resources from the endosperm necessary for germination. Abscissic acid (ABA) shows an accumulation pattern complementary to the GAs, being the main hormone that inhibits all the processes induced by GAs.

Briefly, the seed development process can be divided into two main phases: (a) morphogenesis, or cellular phase, and (b) maturation (Figure 1). Morphogenesis covers all the processes including formation and structural development of the different compartments of the mature seed. In this stage the resources that provide the accessible food reserve for the embryo are also distributed and allocated. The mechanisms that lead to the definition of the structures composing the seed are highly coordinated and extremely complex. They involve a tight hormonal control and a continuous interchange of signals *from* and *to* the maternal tissues, and *between* the two major seed compartments, embryo and endosperm. The incessant communication among the three parts composing the seed will ensure its coordinated development.

Maturation is the physiological process that ends with the onset of the state of seed dormancy. In this stage the seed loses up to 95% of its water content (desiccation), nutrients are stored in the endosperm (Monocots) or in the cotyledons (Eudicots), cell cycle activities are stopped, RNA and protein synthesis decrease (Sheridan and Clark, 1987; Goldberg et al., 1989, 1994; Raz et al., 2001). Embryo growth during maturation is exclusively characterized by events of cellular expansion without cell divisions, and subsequently cell differentiation. During late maturation the seed is metabolically quiescent and highly tolerant to hydric stress (state of dormancy).

The study of plant embryogenesis and seed development has been facilitated by the characterization of mutants (Parcy and Giraudat, 1997; Gazzarrini et al., 2004; Yang et al., 2008; Pignocchi et al., 2009; Xing et al., 2013). Thanks to functional analysis of mutants and misexpression experiments some of the genes and “signals” affecting seed development have been discovered (Garcia et al., 2003; Luo et al., 2005; Ohto et al., 2005, 2009; Chourey et al., 2006; Wang et al., 2010a). In addition, QTL mappings allowed the identification of several loci with significant effect on seed weight and size (Orsi and Tanksley, 2009). Nevertheless, the molecular mechanisms that control the transition into the maturation phase and those that precede cell growth and division are not yet fully elucidated.

Many of the studies on seed development use *Arabidopsis thaliana* as model plant for the Eudicots and *Zea mays* L. for the Monocots. Despite the fact that Monocots and Eudicots share the same seed structures, the processes that lead to seed development and maturation reveal remarkable differences between the two groups.

In this review, we discuss the relevance of the communications between the three compartments of the seed during its development, by a comparative analysis of the latest findings in Arabidopsis and maize. We will summarize the elements that participate in the “flow” of signals that control seed development, and describe in more detail the regulation of this process exerted by the phytohormones, particularly by auxin.

## The process of seed development in arabidopsis

### Establishment of the seed compartments

The process of seed formation, development and maturation of Arabidopsis plants has been well described in several recent reviews (Becraft and Asuncion-Crabb, 2000; Berger, 2003; Olsen, 2004; Santos-Mendoza et al., 2008; Sun et al., 2010). Soon after fertilization, the endosperm nuclei undergo successive mitotic divisions without cell wall formation, generating the multinucleate endosperm, or coenocyte (pre-globular stage) (Figure 1A). This phase is followed by cellularization of the endosperm, and the definition of three regions: the micropylar, the peripheral and chalazal endosperm (Sorensen et al., 2002). As the embryo sac expands, the central vacuole enlarges displacing the cytoplasm of the endosperm to a peripheral position. The cellularized endosperm acts as nourishing tissue that is consumed by the embryo during maturation. The main storage products (lipids and proteins) accumulate in the profusely grown cotyledons. In mature seeds, the embryo fills the seed volume, while a single peripheral endospermic cell layer persists. It contains only a few storage products and the function of these cells is important during seed dormancy, germination and seedling nourishment (Bethke et al., 2007; Holdsworth et al., 2008).

### Importance of communication between the seed compartments

A strong interdependent relationship has been described among seed compartments (Nowack et al., 2010). A failure in the development of one of these compartments, or in the “communications” through their structures, will cause defects in the mature seed, and in some cases even abortion and embryo death. The characterization of mutants presenting phenotypes affecting seed development has helped to unravel some of the communication pathways between seed structures (Chaudhury and Berger, 2001; Berger, 2003; Berger et al., 2006; Nowack et al., 2010).

Mutations affecting endosperm formation at different stages have been described. For instance, knockout mutants such as *short hypocotyl under blue1* (*shb1*), *miniseeds3* (*mini3*), and *haiku1* and *2* (*iku1* and *2*) display a reduced seed size due to alterations in the cellularization of the endosperm. SHB1 binds to the promoters of *MINI3* and *IKU2*, which act in the same pathway to induce their expression and trigger endosperm proliferation and seed growth (Kang and Ni, 2006). These mutants also display a limitation of cell elongation in the surrounding integuments. Thus, the effect of the endosperm on the development of the seed coat points out the relevance of the communication between these two compartments. The reduced seed size in *mini3, iku1*, and *iku2* knockout mutants has also recently been associated with a reduced triacylglycerol content in the embryo (Fatihi et al., 2013).

Genetic evidences revealed the influence of the maternal tissues on endosperm development, exerting their control through chromatin remodeling mechanisms (Drews et al., 1998; Grossniklaus et al., 1998; Chaudhury and Berger, 2001).

Furthermore, it has been shown that endosperm development is required for proper embryo development (Berger, 2003; Berger et al., 2006). Mutations affecting endosperm development (Portereiko et al., 2006; Bemer et al., 2008), cellularization (Pignocchi et al., 2009) or breakdown (Waters et al., 2013a) affect, interrupt or even prevent embryo development and growth. A failure in endosperm cellularization or development will also affect the amount of nutrients stored in the seed, which are essential for embryo maturation. One of the nutrients that largely affects the progression of embryo development is sucrose (Ruan et al., 2010). APETALA2 (AP2), which is a floral patterning regulator, together with *At*SUC5 (Baud et al., 2005) is involved in the control of sucrose ratio and seed mass. *ap2* mutant displays alteration in seed size leading to a bigger seed compared to the wild type (Jofuku et al., 2005). In addition to sugars, the growth-promoting phytohormones cytokinins, brassinosteroids, and auxins are considered important signaling molecules in seed development (Sun et al., 2010). A major role for brassinosteroids and auxins in the control of seed size has been elucidated (Schruff et al., 2006; Martinez-Andujar et al., 2012; Jiang et al., 2013; Jiang and Lin, 2013). An overview of the specific role of each of these hormones in seed development will be given later in this review.

Finally, *LEAFY COTYLEDON1* (*LEC1*), *LEC2*, and *FUSCA3* (*FUS3*) are genes principally expressed in the embryo, and required to maintain cell fate. Nevertheless, it has recently been shown that, toward the phase of maturation, the expression of these genes is also detectable in the endosperm. Thus, their function is determinant for both embryo development and for initiation and maintenance of the maturation phase. Unsurprisingly, alterations in their expression cause dramatic effects on seed phenotype (Bäumlein et al., 1994; Meinke et al., 1994; Lotan et al., 1998; Gazzarrini et al., 2004).

## The process of seed development in maize

Maize is widely considered the model plant for studies regarding seed development in Monocots. The program of seed development in Monocots is basically the same as that in the Eudicots; from the double fertilization event to the endosperm cellularization the processes are highly conserved (Brown et al., 1996a,b) (Figure 1). The main difference between the two models is in the later stage of endosperm development. While in Arabidopsis the endosperm is absorbed at the end of the maturation phase to provide space for the embryo to grow, in maize the endosperm persists and covers other important roles on embryo development and seed organization.

After cellularization of the coenocyte four main cell types differentiate and characterize the fully developed endosperm: the basal endosperm transfer layer (BETL) or transfer cells, the aleurone layer, the starchy endosperm and the cells of the embryo-surrounding region (ESR) (Figure 1B). The transfer cell domain localizes in the basal part of the endosperm. It derives from three initial cells in the chalazal region of the endosperm coenocyte that after division assume transfer cell identity (Figure 1B). The function of the BETL is to load and distribute the nutrients coming from the maternal tissues to the endosperm. Genes expressed at the early stages of the transfer cell differentiation are of special interest to understand how these cells are specified. Many of them are involved in signal transduction and/or transcriptional regulation between maternal tissue and developing seed recently reviewed in Lopato et al. (2014). The aleurone layer delimitates the transfer cell region and constitutes the outer layer of cells of the endosperm. It represents the region of separation between the seed coat and the starchy endosperm. Aleurone cell differentiation occurs exclusively in response to surface position and does not involve specific maternal signals input (Gruis et al., 2006; Reyes et al., 2010). However, recent studies showed that some phytohormones have a prominent role in the determination of aleurone cell fate (Geisler-Lee and Gallie, 2005; Bethke et al., 2006; Forestan et al., 2010). The specification of the aleurone cells also depends on the expression of specific genes such as *Crinkly4* (*Cr4*) (Becraft et al., 1996) and *Defective in kernel1* (*Dek1*) (Becraft and Asuncion-Crabb, 2000; Lid et al., 2002, 2005). Interestingly, *dek1* mutant specifically lacks the aleurone cell layer, but still maintains the transfer cells, supporting the idea that aleurone and transfer cells originated from different specification processes (Lid et al., 2002). A large number of mutants with defects in endosperm and embryo have been described (Neuffer and Sheridan, 1980; Sheridan and Neuffer, 1980). In the *defective kernel* (*dek*) mutants, both embryo and endosperm development are generally altered, while defective endosperm (*de*) mutants show alterations in endosperm development (described in Manzocchi et al., 1980; Pasini et al., 2008). A subclass of *dek* mutants is represented by the *empty pericarp* (*emp*) mutants (Scanlon et al., 1994), which display reduced endosperm and pericarp loss. In these mutants the defects in embryo organization seem to be the origin of the compromised endosperm, pointing out, as in Arabidopsis, the importance of communication between the seed compartments. After seed maturation, the aleurone cell layer participates in the process of seed germination by synthesizing the enzymes that hydrolyze the resources stored in the endosperm and by constituting, together with the embryo, a source of oil storage (Saoussem et al., 2009).

The starchy endosperm is the largest part of the seed in which starch and proteins accumulate serving the embryo germination. It originates from the inner cell generated in the first periclinal cell division of the endosperm, in which the external cell will generate the aleurone. *dek1* and *cr4* mutants curiously maintain the starchy endosperm and replace the aleurone layer with starchy cells.

The ESR is the cavity of the endosperm where the embryo develops. It supplies the nutrients and constitutes the route of communication between the embryo and the surrounding endosperm.

The characterization of seed structures formation in maize, and the genetic dissection of the process of seed development have been mainly performed by a mutational approach. Complementary to the study of mutants, advanced techniques of molecular biology offer nowadays the possibility to investigate all those cases in which the analysis of certain mutations would not be possible. For instance, the cases in which the defects in embryo/seed structures are as severe as to cause lethality. The identification of these mutations by techniques of deep sequencing provides information about gene identity and its implication in the process that has been interrupted or disturbed by the mutation. Recently, Lu et al. (2013) and Sekhon et al. (2014) made important contributions to the study of maize seed development. In their works they analyzed gene transcription by RNA-sequencing and obtained detailed information about differential gene expression between embryo and endosperm. Transcripts were classified, organized in subgroups and several interesting regulatory networks between the two compartments were proposed.

## Hormonal coordination of seed development

Developing seeds consist of multiple tissues and cells with specific patterns of proliferation and differentiation. In order to integrate and organize cell distribution within the tissue/organ, determine cell fate, and control the progression through development, a precise spatial and temporal coordination is required. Cells are able to control all these activities through the production and perception of “signals.” The transmission and perception of these signals is important not only among seed structures, but also within the same compartment to control the progression of the developmental process. Hormones constitute part of these signals (Figure 1C).

In the next sections, we summarize the current knowledge about the mechanisms of communication between the three structures of the seed, especially through the hormonal route. We cover the process of seed development from fertilization of the ovule to maturity. While other scientists have reviewed several mechanisms governing the signaling between the three seed compartments (Nowack et al., 2010), in this review we will focus on the hormonal control of seed development, especially emphasizing the role of auxin.

### The role of auxin in arabidopsis and maize development

Auxin is a key hormone for plant growth and development, accomplishing important roles during the entire lifespan of the plant (Tanaka et al., 2006; Teale et al., 2006; Vanneste and Friml, 2009). It has been shown to be fundamental in the first steps of seed development, as well as for the determination of embryo structure and size (Hamann et al., 2002; Friml et al., 2003; Jenik and Barton, 2005; Cheng et al., 2007; Wabnik et al., 2013). In Arabidopsis it has been demonstrated that auxin plays a role in seed dormancy and germination through its crosstalk with other hormones such as Abscissic acid (ABA) (Liu et al., 2007a, 2013).

In Arabidopsis and maize correct seed development requires a coordinated crosstalk between the seed tissues (mainly embryo and endosperm). One of the most important signaling molecules involved in this communication is auxin. Auxin accumulation and distribution varies during seed development. In fact, the use of auxin marker tools revealed that, at the beginning of development, in immature Arabidopsis seeds, auxin accumulates in the embryos, concretely at the root apex, ends of cotyledon primordia and at the hypophysis (Ni et al., 2001). In maize, auxin concentration increases at the onset of endoreduplication and remains high throughout the development (Lur and Setter, 1993b). More specifically, it has been shown that auxin is low during the initial phase of endosperm development and increases from 9 to 11 days after pollination (DAP) remaining high until maturation (Lur and Setter, 1993a,b). In a recent work by Bernardi et al. (2012) it was shown that free indole-3-acetic acid (IAA) levels increase between 8 and 28 DAP with a drop at 20 DAP. These results suggest a role for auxin in all the stages of maize seed development. Auxin is involved in positional signaling during aleurone development and specification (Forestan et al., 2010). Complete loss of endogenous auxin in the embryo might be lethal, confirming a key role of this phytohormone in embryo development and germination.

The mechanism of action of auxin involves three checkpoints: biosynthesis, polar transport, and perception/transduction of the signal. These three main processes, which will be extensively discussed in the next sections, are involved in both maize (Forestan and Varotto, 2012) and Arabidopsis seed development, influencing the final size.

#### IAA biosynthesis

The first natural auxin identified was the IAA, which originates from its precursor, the amino acid tryptophan (Trp). Five IAA biosynthetic pathways have been proposed: four interconnected Trp-dependent IAA biosynthetic pathways, and a Trp-independent pathway (Tivendale et al., 2014). Recently, it has been discovered that YUC (flavin-monoxygenases) and TAA/TAR (tryptophan amino-transferases), two of the most relevant enzymes involved in the biosynthesis of auxin, act in the same pathway, the so-called indolic 3-pyruvic acid (IPA) pathway (Mashiguchi et al., 2011; Stepanova et al., 2011; Won et al., 2011). Based on these studies, it was proposed that YUCCA (YUC) proteins catalyze the rate-limiting step of the IPA pathway. Counts of 11 *YUC* genes have been made in the Arabidopsis genome (Zhao et al., 2001; Cheng et al., 2006); four of them, *YUC1, YUC4, YUC10*, and *YUC11*, being expressed in an overlapping way in the embryo (Cheng et al., 2007). While the double mutant *yuc1 yuc4* does not show any obvious phenotype during embryogenesis, the quadruple mutant *yuc1 yuc4 yuc10 yuc11* displays several morphological defects, already at the embryonic globular stage, failing also in developing hypocotyls and primary roots. This strong phenotype is similar to that displayed by the mutants in auxin signaling (*monopteros, mp*; *bodenloss, bdl*), perception (*transport inhibitor response1* (*tir1*) *auxin signaling F box protein 1* (*afb1*) *afb2 afb3* quadruple mutant) and transport (*pin-formed 1* (*pin1) pin3 pin4 pin7* quadruple mutant) that will be further described in this review, which indicates that the auxin synthetized by YUC is critical for embryogenesis (Cheng et al., 2007).

It has been found that the modulation of the auxin biosynthetic genes (i.e., *YUC1, 2, 4*, and *10*) is controlled by transcription factors such as LEC2 during somatic embryogenesis and particularly, it was described that *YUC4* is a direct target of LEC2 (Stone et al., 2008; Wojcikowska et al., 2013). This indicates that regulation of the expression of key auxin biosynthetic genes by transcription factors is one of the mechanisms that modulate the hormone levels in Arabidopsis.

In maize, the seed is the organ that accumulates the greater content of IAA and most of the free auxin is synthesized *in situ* by the Trp-dependent auxin pathway. Compared to the 11 *YUC* genes identified in Arabidopsis, only four *YUC-like* genes have so far been identified in maize (Bernardi et al., 2012), (Table 1). The first evidence of the relevance of TAR and YUC function in maize emerged from two parallel works based on the study of the expression pattern of these genes in *mn1* mutant. This mutant displays a reduced IAA content in the seed, and despite a higher expression of *ZmTAR* than *ZmYUC1* (in both mutant and wild type), only the *ZmYUC1* showed a reduced level of expression in the mutant, suggesting a key role of this gene in auxin biosynthesis (Chourey et al., 2010; Le Clere et al., 2010). In maize, three of the *TAR* orthologs are highly expressed in the endosperm (Bernardi et al., 2012), while of the four *YUC* orthologs, only *ZmYUC1* is mainly expressed in this tissue (Chourey et al., 2010; Le Clere et al., 2010), and it was reported recently that its expression correlates with IAA accumulation (Bernardi et al., 2012). Moreover, it was observed that in order to obtain an altered seed phenotype in Arabidopsis it is necessary to produce a quadruple *yuc* mutant (as described in the first part of this section); however, in maize the single mutation in *ZmYUC1* is sufficient to cause alteration in seed phenotype (Bernardi et al., 2012). This observation permits to speculate that, with respect to Arabidopsis, the *YUC* genes of maize show a higher tissue-specificity, while they are largely redundant in Arabidopsis.

**Table 1:** **Genes involved in the hormonal control of seed development in Arabidopsis and maize**.

**Gene name**	**Acronym**	**Biological function of the encoded protein**	**Seed compartment expression**	Species^*^
*YUCCA 1*	*YUC1*	Key enzymes of tryptophan-dependent auxin biosynthesis. Function in seed development and morphogenesis	Embryo, Endosperm,	A
*YUCCA 4*	*YUC4*		Seed coat	
*YUCCA 10*	*YUC10*			
*YUCCA 11*	*YUC11*			
*PINFORMED 1*	*PIN1*	Auxin efflux carriers involved in early embryo development. Establish the apical-basal auxin gradient	Embryo	A
*PINFORMED 3*	*PIN3*			
*PINFORMED 4*	*PIN4*			
*PINFORMED 7*	*PIN7*			
*Transport Inhibitor Response 1*	*TIR1*	Part of the SCF E3-ubiquitin ligase complex. Functions as auxin receptor and is responsible for auxin signal transduction	Embryo, Endosperm	A
*AUXIN SIGNALING F-BOX 1*	*AFB1*	F-box proteins that form a complex with TIR1. Involved in regulation of auxin response	Seed coat (*AFB2* and *AFB3*) and Embryo	A
*AUXIN SIGNALING F-BOX 2*	*AFB2*			
*AUXIN SIGNALING F-BOX 3*	*AFB3*			
*MONOPTEROS/*	*MP/ARF5*	Transcriptional activator. Regulates embryo development	Embryo	A
*AUXIN RESPONSE FACTOR 5*				
*BODENLOSS/*	*BDL/*	Transcriptional repressor. Interacts with MP preventing it from activating its targets	Embryo	A
*Auxin/INDOLACETICACID 12*	*Aux/1AA12*			
*ETTIN/AUXIN RESPONSE FACTOR 3*	*ETT/ARF3*	Controls the integument development	Seed coat	A
*ABERRANT TESTA SHAPE*	*ATS*	Forms a complex with ETT. Involvement in integument formation	Seed coat	A
*MEGAINTEGUMENTA/AUXIN RESPONSE FACTOR 2*	*MNT/ARF2*	Regulates seed size. Interacts with BIN2. Growth repressor	Seed coat, Embryo	A
*ZmYUCCA 1*	*ZmYUC1*	Involved in auxin biosynthesis in maize endosperm. Controls seed size	Endosperm	M
*Sparse inflorescence1*	*Spi1*	A YUCCA ortholog in maize. Role in maize inflorescence development	Embryo	M
*Vanishing tassel 2*	*Vt2*	A TAA ortholog in maize. Role in vegetative and reproductive development	Embryo	M
*Orange pericarp 1*	*orp1*	Both *orp1* and *orp2* encode the *beta* subunit of tryptophan synthase. Required for seedling development	Embryo	M
*Orange pericarp 2*	*orp2*		Endosperm	
*Miniature 1*	*Mn1*	Cell wall invertase. Role in nutrient allocation and crosstalk with auxin	Endosperm	M
*ZmPINFORMED 1*	*ZmPIN1*	Auxin efflux carriers involved in polar transport during embryogenesis and endosperm formation	Embryo	M
*ZmPINFORMED 2*	*ZmPIN2*		Endosperm	
*ZmPINFORMED 5*	*ZmPIN5*			
*ZmPINFORMED 8*	*ZmPIN8*			
*ZmPINFORMED 10*	*ZmPIN10*			
*SEMAPHORE1*	*SEM1*	Regulator of *knox* gene expression. Required for proper kernel development	Embryo	M
			Endosperm	
*ABERRANT PHYLLOTAXY 1*	*ABPH1*	Cytokinin-inducible type A response regulator. Negative regulator of SAM size and positive regulator of *PIN1* expression	Embryo	M
*Histidine phosphotransfer proteins*	*AHPs*	Cytokinin signal transducers. Regulate seed size	Endosperm, seed coat	A
*Histidine Kinase*	*AHK*	Cytokinin receptor. Regulates seed size	Seed coat	A
*Response Regulators*	*ARRs*	Targets of the AHPs. Together with cytokinin response proteins regulate endosperm development	Endosperm	A
*SHRINK/CYP72C1*	*SHK1*	Decreases brassinosteroids levels. Regulates cell division and seed size	Embryo Endosperm, Seed coat	A
*CITOKININ OXYDASE 1 CITOKININ OXYDASE 2*	*CKX1 CKX2*	Regulate seed size and weight	Endosperm	A
*CITOKININ OXYDASE 3*	*CKX3*			
*DWARF 5*	*DWF5*	Endoplasmic reticulum transmembrane protein involved in brassinosteroids signaling	Embryo, endosperm, seed coat	A
*ZmHistidine kinase*	*ZmHK1*	Cytokinin receptor-like genes. Control seed size	Embryo	M
	*ZmHK1a2*			
	*ZmHK2*			
	*ZmHK3b*			
	*ZmHK2a2*			
*DE-ETIOLATED 2*	*DET2*	Gene of brassinosteroids biosynthesis. Controls embryo development, seed size and embryo cell number	Embryo, Endosperm	A
*BRASSINOSTEROIDS INSENSITIVE 1*	*BRI1*	Protein kinase, regulate brassinosteroids and phosphorilatesARF2. Growth Repressor	Seed coat, Endosperm	A
*BRASSINOSTEROIDS INSENSITIVE 2*	*BIN2*			
*BRASSINAZOLE-RESISTANT 1*	*BZR1*	Positive brassinosteroid-signaling protein. Phosphorylated by BIN2	Endosperm	A
*ZmStarch synthase I*	*ZmSSI*	Starch synthase induced by ABA	Endosperm	M

*Only the genes mentioned in the text are showed*.

* *A, Arabidopsis; M, Maize*.

Several mutants defective in auxin biosynthetic genes were identified in maize, i.e., *orange pericarp* (Wright et al., 1992); *vanishing tassel2* (Phillips et al., 2011) that is homologous to *TAA1*; *sparse inflorescence1* (Gallavotti et al., 2008) and *defective endosperm18* (Torti et al., 1986; Bernardi et al., 2012) both homologous to *AtYUC*. Of these, the only mutant showing defective phenotype in seed is *de18*. The knockout mutation of *ZmYUC1* in the mutant *de18* still retains a high level of TAR despite a decrease in IAA during early endosperm development (1-7% respect to wild type) suggesting that TAR and YUC may act on the same pathway also in maize seed (Bernardi et al., 2012).

The *orange pericarp* (*orp*) mutant, which is defective in Trp-synthesis originating from indole, produces plants that are seedling lethal that can be partially rescued by supplying Trp, and accumulates indole and anthranilate (Wright et al., 1992). The fact that this mutant still produces auxin provided the first evidence that also a Trp-independent biosynthesis occurs in maize.

#### Auxin polar transport

**Figure 2:**
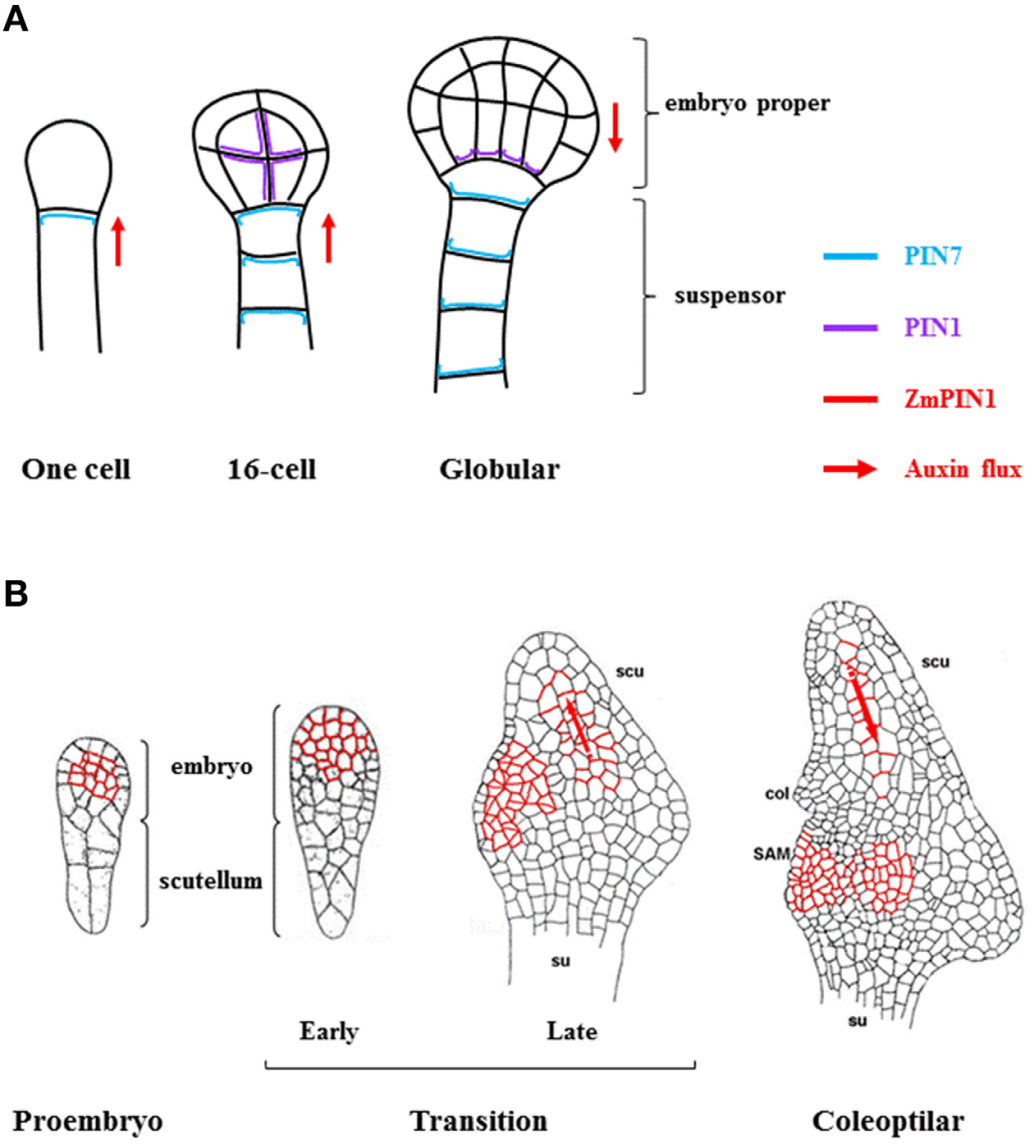
**Auxin transport during the embryogenesis development of Arabidopsis and maize. (A)** Schematic representation of auxin transport during embryo development in Arabidopsis. In early embryo (one-cell stage to 16-cell stage in the figure), PIN7 (blue) is expressed in the suspensor cells localizing to the apical membranes, mediating auxin transport toward the proembryo. During the octant (not shown) and 16-cell stage, all proembryo cells express PIN1 (purple), which is evenly distributed along the inner cell membranes and not polarly localized. Later, during the transition to the globular stage, the subcellular localization of PIN1 becomes polar, facing the basal membranes. Simultaneously and similarly, the polarity of PIN7 localization is reversed, now localized at the basal membrane of suspensor cells. The localization of PIN1 and PIN7 in the basal membranes establishes an apical-basal flux of auxins that will be maintained throughout the life cycle of the plant (adapted from Friml et al., 2003; Weijers and Jurgens, 2005; Nawy et al., 2008). **(B)** Model for the *Zm*PIN1-mediated auxin transport during early stages of maize embryogenesis. Medial longitudinal sections of maize embryos at proembryo, transition and coleoptilar stages are shown. The *Zm*PIN1 protein (red) localizes in embryo plasma membranes. After the first division of the zygote, several cell divisions in different planes lead to the formation of the small embryo and the larger suspensor (proembryo stage). At this stage, auxins accumulate in the endosperm above the embryo but not in the embryo itself (not shown), and *Zm*PIN1 localizes at the cell boundaries of the undifferentiated proembryo core, without any polarity. Later, adaxial/abaxial polarity is established by the outgrowth of the scutellum at the abaxial side of the embryo (early transition stage). *Zm*PIN1 is polarly localized in the apical anticlinal membranes, marking the provascular cells of the differentiating scutellum, indicating an auxin flux toward the tip of the single maize cotyledon (late transition stage). At the coleoptilar stage there is the switch from apical to basal gradient of *Zm*PIN1, followed by a change of the auxin flux (adapted from Forestan et al., 2010; Chen et al., 2014). col, coleoptile; SAM, shoot apical meristem; scu, scutellum; su, suspensor. In both A and B the red arrows indicate the auxin efflux mediated by PINs.

The role exerted by auxin in the regulation of plant growth strongly depends on its characteristic polar transport. Plants have evolved a unique mechanism of directional cell-to-cell transport of this growth regulator, determinant for the generation of a polarized embryonic axis. The relevance of the apical-basal axis establishment is that it will determine the body plan of the adult organism. Auxin transport is realized by the PIN efflux transporters, the auxin influx carriers (AUX/LAX1 family) and the PGP proteins belonging to the ABCB transporter superfamily (Bennett et al., 1996; Petrasek et al., 2006). The polar subcellular localization of the carriers (influx/efflux) establishes the directional flow of auxin. In Arabidopsis there are eight genes encoding PIN proteins (*PIN1-8*), of which only *PIN1, PIN3, PIN4*, and *PIN7* are expressed in the embryo. Relocation of PIN1 and PIN7 has been shown to be crucial for embryo polarity establishment (Friml et al., 2003). Although the upstream mechanisms directing the polarization of auxin during embryogenesis are still practically unknown, it was shown that 24-h after fertilization auxin peaks in the funiculus, the chalaza, and the micropyle of the ovule (but not in the valve), which indicates that the increase in auxin levels in the young seeds is probably due to a maternal origin (Dorcey et al., 2009). Friml et al. (2003) described through the analysis of the *DR5rev::GFP* marker line (a synthetic promoter that responds to auxin response factors) an accumulation of auxin in the apical cell of the embryo, just after the first divisions of the zygote, while a weak signal was detected in the suspensor. Later on during development, auxin signaling is localized in the upmost suspensor cells. At later stages of embryogenesis, auxin signal is detected at the tips of the developing cotyledons and provascular veins. Thus, auxin seems to determine the division patterns and specification of the cells derived from the zygote, and this pattern is determined by the activity of the PINs. PIN1 and PIN7 are required in the process of early embryo development. In fact, their asymmetric subcellular localization has been considered responsible for controlling polar auxin flow in post-embryonic development (Friml et al., 2003), (Figure 2A). PIN7 is localized in the apical membranes of suspensor cells, while PIN1 localizes in pro-embryo cells, first in a non-polarized manner, while later, at the globular stage, it is recruited to the basal membrane. PIN7 polarity is then reversed, facing the basal membrane of suspensor cells. The polarization of PIN1 and reversion of polarity of PIN7 correlate with an apical-basal reversion of the auxin gradient (Friml et al., 2003). The biological function of the *PIN*s in Arabidopsis seed development has been clarified by analyzing the phenotype of their mutants. A high percentage of embryos in *pin7* mutant do not correctly establish the apical-basal auxin gradient. Morphologically, the mutant embryo shows defects at the proembryo stage resembling those of *mp* and *bdl* mutations (see details below), bearing filamentous structures at late stages of embryo development. However, in many cases *pin7* mutants recover at globular stage, corresponding to the moment when PIN1 protein starts to be localized at the basal part of the cellular membrane and *PIN4* expression increases. *pin1* mutants also display defects at the basal embryo pole. Higher order mutants were generated by combining *PIN*s single mutants, specifically expressed in the embryo (*pin1, pin3, pin4*, and *pin7*). The severity of the phenotypes increased additively as higher order mutants were generated. The quadruple mutant *pin1 pin3 pin4 pin7* is the combination that displayed the most severe phenotype. These mutants, depending on the genetic background of the ecotype, can manifest embryo lethality or, when the embryo survives, will generate a plant with severe apical defects and non-functional or absent roots. The severity of the phenotype showed by these multiple mutants indicates the existence of some functional redundancy among the different PINs in the embryo (Friml et al., 2003).

In the seed of maize, a gradient of auxin might be responsible for a correct differentiation of both embryo and endosperm (Forestan et al., 2010). The switch from the apical to basal membrane localization of *Zm*PIN1 proteins characterizes the coleoptilar stage and the following establishment of an auxin flux from both the differentiated scutellum and the shoot apical meristem (SAM) that is responsible for the differentiation of embryonic roots. The final stage, in which auxin polar transport is involved during embryogenesis, is the formation of leaf primordia, suggested by *Zm*PIN1 localization in the subapical region of the meristem (Forestan et al., 2010) (Figure 2B).

Among the auxin transport carrier genes known in Arabidopsis, the PIN family of efflux carriers has been extensively studied in maize (reviewed in Forestan and Varotto, 2012). In particular, 12 *PIN* genes, 2 *PIN-like* and 1 *ABCB* were identified (Forestan et al., 2012). *ZmPIN1, ZmPIN2, ZmPIN5*, and *ZmPIN10* had overlapping expression during early seed development and their transcripts were localized in different subcellular domains. *ZmPIN8*, which was up-regulated in all the seed stages analyzed, was expressed in BETL cells, maternal chalazal tissues and aleurone layer (Forestan et al., 2012). In the same work it was proposed that some *ZmPIN* genes could be subjected to sub-functionalization. Some conserved mechanisms in Arabidopsis and maize are found during embryogenesis where *ZmPIN1a-c* appeared to have a redundant function as previously reported for Arabidopsis *PIN* genes (Vieten et al., 2005) (Figure 2).

In a recent work by Chen et al. (2014) it was shown that the auxin signaling from the endosperm was important to pattern embryo development during early embryogenesis in maize. More specifically, the auxin signal detected at the surface of the adaxial embryo was correlated to the specification of embryo proper, SAM and scutellum. As previously mentioned, the *Zm*PIN1 signals were polarized first in the apical and in the basal membrane of epidermal L1 cell layer suggesting an auxin flux from these cells to the inner layers (also detailed in Figure 2). Finally, the same authors suggested a new function for the ESR during the early stages of development, being responsible for preventing the auxin flux from the endosperm to the embryo.

Concerning the endosperm specification, a gradient drop off specifies the aleurone fate maintaining the outer layer above a specific IAA threshold. Indeed, in normal maize endosperm, the auxin concentration is high at the endosperm margin and lower in the center. In the presence of NPA, an auxin transport inhibitor, auxin accumulates above this threshold in many cell layers, resulting in a multilayered aleurone (Becraft and Asuncion-Crabb, 2000; Becraft and Yi, 2011). A further mutant that shows a reduction of auxin transport is *semaphore1* (*sem1*). *sem1* shows a dwarf phenotype with defects also in endosperm and embryo patterning (Scanlon et al., 2002) but the gene mutation responsible for the alteration in the polar auxin transport has not yet been identified.

#### Signal transduction

At the molecular level, auxin response is mediated by the action of AUXIN RESPONSE FACTORS (ARFs). The ARFs are transcription factors that recognize specific sequences termed Auxin-Response Elements (AuxREs) present in the promoter of auxin-responsive genes, activating or repressing their transcription (Abel and Theologis, 1996; Ulmasov et al., 1999). However, the ARFs do not seem to be able to regulate gene expression in response to auxin by themselves, but require interaction with the AUXIN/INDOLE-3-ACETIC ACID (AUX/IAA) proteins, which constitute the repressors of auxin signaling (Kim et al., 1997). Aux/IAAs function as transcriptional repressors by binding and sequestering the ARFs. The degradation of the Aux/IAA proteins is induced by auxin (Guilfoyle and Hagen, 2007). Briefly, the F-box protein Transport Inhibitor Response 1 (TIR1), which has been identified as an auxin receptor (Dharmasiri et al., 2005; Kepinski and Leyser, 2005), interacts with the E3-Ubiquitin ligase Skp1/Cullin/F-box (SCF) complex, generating the SCF^TIR1^ complex, and determining the substrate specificity toward the Aux/IAA proteins. These proteins are thus ubiquitinated, and marked as substrates for proteasomal degradation (Tan et al., 2007). Dharmasiri et al. (2005) described three additional genes, *AFB1, 2*, and *3*, which encode F-box proteins also interacting with the SCF-complexes. In Arabidopsis, *TIR1* and *AFB1* are moderately expressed during embryogenesis, while *AFB2* and *3* expression is high. The generation of high order mutants for the four genes resulted in a progressive decrease in auxin response, as well as an increase in the severity of the defects in development. The severe effect of the quadruple mutation is manifested in the lack of root, the disappearance of hypocotyl, and often by the formation of a single cotyledon resembling *bdl* or *mp* mutants.

Genome-wide analyses on the maize reference genome were performed to identify the *Aux/IAA* (Wang et al., 2010b) and *ARF* gene families in maize (Liu et al., 2011; Xing et al., 2011; Wang et al., 2012). The 31 identified *ZmAux/IAA* genes showed a putative expression (based on EST mining) in different tissues and organs that suggests a temporal and spatial pattern of regulation. The 31–36 *ZmARF* are also known to have a tissue-specificity that changes during plant development (Wang et al., 2010b, 2012; Liu et al., 2011; Xing et al., 2011). Each *ZmARF* possesses a sister pair due to chromosomal duplication of the maize genome, and they are distributed in all the chromosomes except chromosome 7 (Xing et al., 2011; Wang et al., 2012). Xing et al. (2011) found that about half of the *ZmARF* genes identified have an auxin responsive element in their promoter regions and 18 were predicted to be targets of small RNAs. Furthermore, in the same work, seven *ZmARF* genes were constitutively expressed in developing embryo suggesting the importance of auxin signaling during embryo formation in maize. Despite the genomic information, there is still a lack of knowledge about the functional role of these auxin-related factors in maize.

In Arabidopsis 23 ARFs and 29 Aux/IAA were identified (Okushima et al., 2005). Of these, only three ARFs and one IAA have been characterized as having a role in seed and embryo development. ARF5/MONOPTEROS (MP) is an activator of auxin-responsive genes and constitutes a key element in the development process of the embryo. While *mp* partial loss-of-function mutants have nearly normal embryo development, their reproductive program is compromised. The phenotype of *mp* strong mutant alleles results in alterations of the basal body region of the embryo, such as malformation of the hypophysis and subsequent absence of the radicle and root meristem, already detectable from the octant stage of embryo development, frequently ending in an embryo lethal phenotype (Berleth and Jurgens, 1993; Hardtke and Berleth, 1998). The gene *BODENLOSS* (*BDL)* encodes IAA12. It was demonstrated that *bdl* mutant, holding a gain-of-function mutation that stabilizes the BDL protein, has a phenotype similar to *mp*. The fact that the double mutant *bdl mp* shows a similar phenotype places BDL and MP in the same pathway (Hamann et al., 2002). In fact, *BDL* is co-expressed with *MP* during early embryogenesis, and it was shown that this protein physically interacts with MP. These results indicate that BDL and MP are IAA-ARF interacting proteins, where in this interaction BDL prevents MP from activating its auxin responsive gene targets. After BDL degradation in response to auxin, the release of MP triggers the correct initiation of the basal body region in early embryogenesis (Hamann et al., 2002).

On the other hand, ARF3/ETTIN (ETT) controls the correct development of the integuments and thus, the seed coat. The *ett* mutant ovules present the same alterations as the *aberrant testa shape* (*ats*) ones, where inner and outer integuments grow together fused in a single wide structure, resulting in rounded, aberrant-morphology seeds, variable in size (Kelley et al., 2012). The authors show that ETT and ATS are able to interact, and propose a model in which, upon integument initiation, ATS-ETT complex accumulates in the ovule, in the abaxial layer of the inner integument, where they negatively regulate *PIN1*, proposing that these two proteins participate together in auxin signaling during seed development (Kelley et al., 2012).

Finally, *megaintegumenta* (*mnt*) mutants, defective in *ARF2*, present larger seeds than wild-type plants due to the formation of a bigger integument (Schruff et al., 2006). Despite the fact that the phenotype of the *mnt*/*arf2* mutant has been already characterized, the ARF2 targets that control seed development are still unknown. In Arabidopsis, the mechanism by which ARF2 is activated has been elucidated and involves a phosphorylation mediated by the protein kinase BRASSINOSTEROID-INSENSITIVE 2 (BIN2), which in turn is regulated by brassinosteroids (Vert et al., 2008). In the *mnt*/*arf2* mutant, seed size and weight are dramatically increased. The enlarged seed coat is due to the presence of extra cells in the integuments before fertilization, generated by extra anticlinal cell divisions. The embryo is also bigger than the wild type, but not the endosperm. Surprisingly for integument mutants, *mnt*/*arf2* does not show female infertility. Given that the *mnt*/*arf2* lesion causes other pleiotropic effects on vegetative and floral development, Schruff et al. (2006) conclude that MNT/ARF2 is a repressor of cell division and organ growth.

#### Nutrient allocation and auxin in maize endosperm

**Figure 3:**
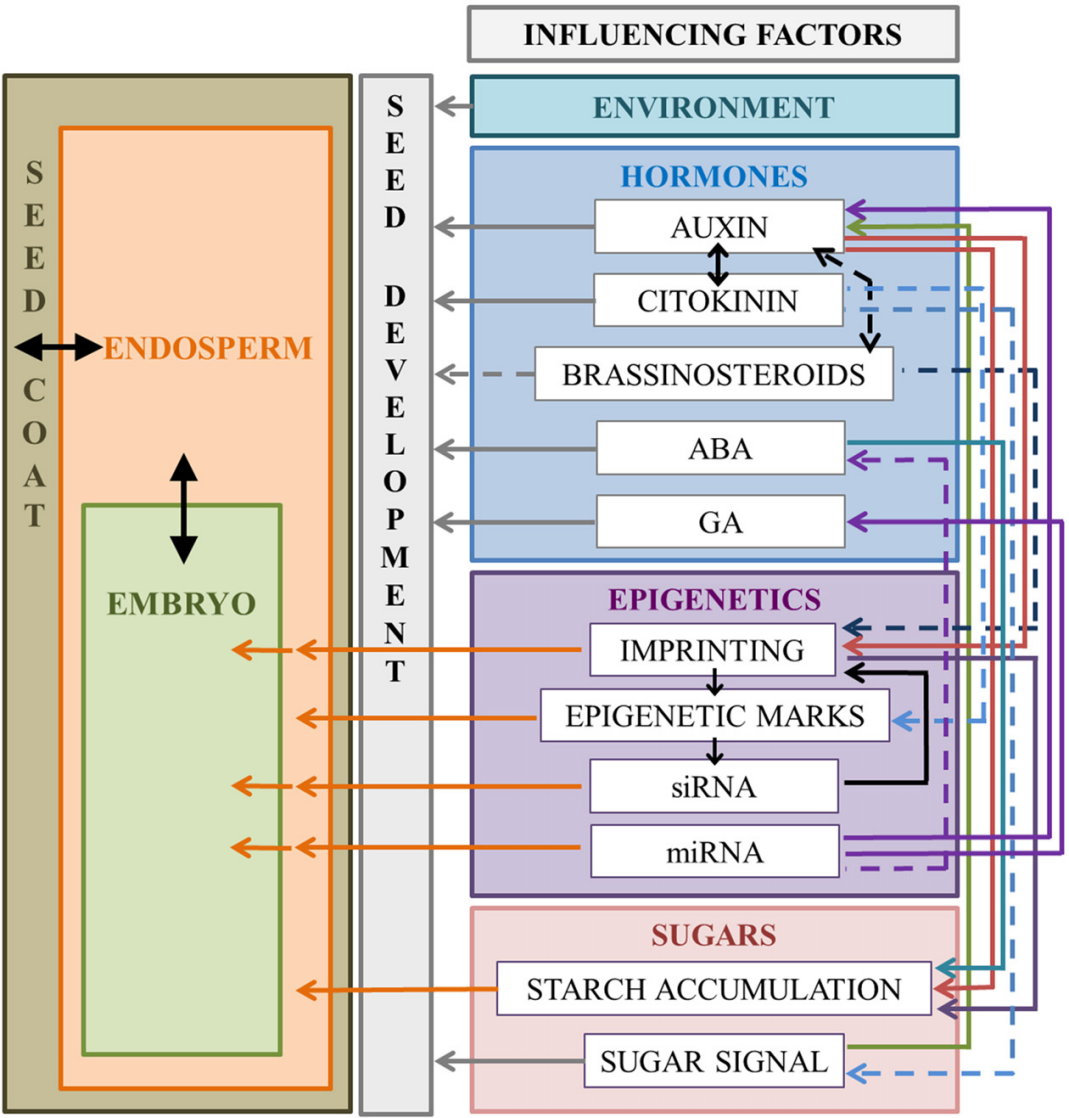
**Schematic representation of the factors affecting seed development in Arabidopsis and maize**. Communication among seed compartments can involve one or more of the factors displayed on the right. The figure shows only the elements cited in this review. Single-headed arrows indicate regulation, double-headed arrows indicate reciprocal influence or regulation, and dashed arrows indicate an effect demonstrated only in one of the two species. Full-headed arrows indicate the communication among seed compartments.

The role of sugars in controlling auxin biosynthesis and metabolism in both maize and Arabidopsis is well known (Le Clere et al., 2010; Sairanen et al., 2012; Figure 3). In maize, auxin-sugar crosstalk involves other aspects peculiar to this Monocot: nutrient accumulation and endoreduplication in the endosperm. The transfer cells or BETL, as the major site of cross-talk between maternal (chalazal) and filial tissue (endosperm and embryo), is essential for nutrient intake and correct endosperm development. Indeed, the altered morphology of BETL is a characteristic of mutants with reduced seed mass such as *de18* (Torti et al., 1984), *mn1* (Kang et al., 2009), and *reduced grain filling1* (Maitz et al., 2000). It has been observed that in the situation of abnormal development of BETL the transport of auxin, and presumably its accumulation, is impaired. Indeed, *de18* mutant exhibits low levels of *ZmPIN1* in the transfer cells with respect to the wild type (Forestan and Varotto, 2010).

Zeins are the main storage proteins of maize kernels, constituting about 70% of total protein content in endosperm. An increase in zein synthesis was observed at both transcript and protein level after exogenous auxin treatment (Lur and Setter, 1993a). In the *defective kernel18* mutant (*dek18)*, that is defective in auxin accumulation, 12 and 14 KDa zeins were found to correlate with auxin content. Indeed, at 20 DAP, these proteins were present in the wild type and in the mutant treated with exogenous auxin and not in the *dek18* mutant (Lur and Setter, 1993b). Whereas the interaction between zeins and auxin has not been proved, the sugar-hormone crosstalk in maize is well known (Chourey et al., 2010; Le Clere et al., 2010). IAA synthesis seems to be required for grain filling in maize and this process is related to the cell wall invertase activity. In *mn1*, which is a small seeded mutant defective in invertase, the low sugar content correlates with the low transcript level of *ZmYUC1* during endosperm development (Le Clere et al., 2010). Indeed, the presence of glucose increased the expression of *ZmYUC1* in cultured kernels (Le Clere et al., 2010). Forestan et al. (2010) showed that IAA accumulation in the three endosperm compartments (BETL, aleurone and ESR) occurs shortly before the starch accumulation phase. The invertase inhibitor ZM-INVINH1 has been reported to bind cell wall invertase during kernel development in maize and is localized to the ESR (Bate et al., 2004). This evidence suggests that invertase activity, together with auxin transport, has a key role in the regulation of events that control carbon partitioning during early kernel development. The evidence that *ZmYUC1* is regulated by hexose sugars, in particular glucose is in agreement with the data reported in Arabidopsis (Hartig and Beck, 2006).

During the early phases of endosperm development, endoreduplication and the synthesis of storage compounds are tightly interconnected (Sabelli and Larkins, 2009). There is limited information on how phytohormones control the endoreduplication in maize. It was shown that the increase of IAA in the late stage of seed development is coincident with the onset of endoreduplication (Lur and Setter, 1993a). Furthermore, analysis of auxin levels in several *dek* mutants indicated a positive correlation between IAA and nuclear size (Lur and Setter, 1993b). The same evidence was found studying *de18* mutant (Bernardi et al., 2012). The low auxin accumulation in the early stages of development of this mutant causes a delay in endoreduplication and a reduced ploidy level with respect to the wild type.

In two recent works about the dissection of the maize seed transcriptome many genes related to hormone metabolism were found enriched, particularly in embryo (Lu et al., 2013; Teoh et al., 2013). Transcription factor analysis on maize seed evidenced that most of the highly expressed genes in the embryo are involved in epigenetic regulation (i.e., methyltransferases and acetyltransferases) and hormone signaling pathways (i.e., ARF and Aux/IAA) (Lu et al., 2013). The role of epigenetics in the control of seed development is briefly discussed in Section Epigenetic Control of Endosperm Development by Genomic Imprinting.

### Role of the other phytohormones on seed development

Although auxin plays a principal role in the regulation of embryo patterning and endosperm development, other hormones have been found to participate in the control of seed development. Their functions in this developmental process and the links between them are briefly discussed in the following sections.

#### The role of cytokinins

The activity of Cytokinins (CKs), together with auxin, is especially linked to growth promotion by cell division, development and differentiation (Bishopp et al., 2011; Vanstraelen and Benkova, 2012). Although the biosynthetic pathway and transmission of the signal are quite well described (Hwang et al., 2012), the function of CKs in the seed has still not been exhaustively characterized. In Arabidopsis, the limited existing knowledge comes from a few reports (Werner et al., 2003; Garcia et al., 2005; Day et al., 2008). Day et al. (2008) identified the *Histidine-containing phosphotransfer proteins* genes (*AHP*s) as the genes preferentially activated by CKs in Arabidopsis. After being phosphorylated by the CKs-receptors (AHKs), the AHP proteins transduce the CK-signal by entering the nucleus and transferring the phosphate group to the Arabidopsis response regulators (ARRs). The ARR proteins constitute a class of regulators in the cytokinin signaling. They comprise the type-A proteins that are normally negative regulators of CKs signaling, and the type-B that positively regulate gene expression. They can directly bind DNA through the MYB-like domain, and contribute to the outputs of the cytokinin signaling through protein-protein interactions by their glutamine (Q)-rich domain. The CYTOKININ RESPONSE FACTOR (CRF) proteins rapidly accumulate in response to CKs. Their function largely overlap with the type-B ARRs, indicating that CRF and ARR belong to a two-component system for the transmission of CKs signaling in the regulation of the development of embryo, cotyledons and leaves (Rashotte et al., 2006). The B-type *ARR* genes (*ARR21, ARR19, ARR18, ARR8*) together with the *CRF2* and *CRF3* genes are preferentially expressed in the endosperm (Day et al., 2008).

Studies of the genes related to CKs production have shown that during the first stages of seed development their expression is principally associated to an effect of the hormone on the development of endosperm and seed coat. These results suggest that the control of seed size would involve a crosstalk occurring between maternal and zygotic tissues (Garcia et al., 2005). Genetic analyses of CK synthetic genes (cytokinin oxidases, *CKXs*) -*AtCKX1* and *AtCKX3*- have indicated that in the corresponding mutants the size of the seed, as well as the embryo, was increased (Werner et al., 2003). Similar phenotypes were found observing the triple mutant of the cytokinin receptors *ahk2 ahk3 ahk4* (Riefler et al., 2006). The effect of an increase in both embryo and seed size was suggested to be under control of maternal and/or endospermal genotypes (Riefler et al., 2006).

Recently, CKs were elucidated to have an important role in the integration of epigenetic and genetic control of seed development. Li et al. (2013) characterized *CKX2* as target of IKU that, as mentioned before, has a relevant role in the promotion of endosperm cellularization, controlling seed size. Several works have been conducted in maize because of the high level of CKs detectable in the seed and the relative ease of study due to the seed size. In most of these studies the relative abundance of the hormone at the early stages of endosperm development and embryo differentiation was reported (Jones, 1990; Lur and Setter, 1993a,b; Dietrich et al., 1995; Brugiere et al., 2003; Veach et al., 2003; Rijavec et al., 2009). Studies on gene transcription in different stages of caryopsis development showed that the genes encoding for enzymes involved in the synthesis of CKs are expressed mainly in endosperm, pedicel and embryo soon after pollination. In the pedicel/placental chalazal/basal endosperm region, CKs levels were 2–3 times higher than in the rest of the seed (Brugiere et al., 2003). At a later stage of seed development, CKs become detectable also in the BETL (Brugiere et al., 2008; Smehilova et al., 2009; Vyroubalova et al., 2009; Rijavec et al., 2011). Immunolocalizations and *in situ* hybridizations showed that CKs are synthesized in certain regions of the seed, but they are also transported from the maternal tissue through the pedicel to the endosperm (Zhang et al., 2009b; Rijavec and Dermastia, 2010). The authors also found that CKs accumulate in the placenta-chalazal cell layer and are able to promote Programmed Cell Death (PCD).

Similarly to Arabidopsis, the main role of CKs detected in the caryopsis of maize is establishing seed size, by promoting endosperm cell divisions. A relationship was elucidated between CKs accumulation and/or activation, and cyclin activity. In respect to Arabidopsis, in which CKs have an effect only on CycD3 activity (Riou-Khamlichi et al., 1999), in maize the CKs affect CycD3 and CycD2 (Gutierrez et al., 2005).

A study on the localization of auxin and cytokinins during early seed development elucidated the role of these two antagonistic hormones. At 6–8 DAP the CKs were detected in both the BETL region and the ESR, while the signal was very low in the embryo (Chen et al., 2014). This evidence correlates with the fast cell division and growth at both apical and basal part of the endosperm. Later in development (9–10 DAP) CKs signal is mainly localized in the epidermis of the scutellum and in the SAM. The contemporary localization of auxin and CKs during early embryo development is different at the scutellum tip, where IAA signal is stronger than that of CKs. In Arabidopsis, transient and antagonistic interaction between auxin and cytokinins is critical for specifying the root-stem cell-pool that will determine the definition of the root-stem axis. Auxin, through a feedback mechanism, is responsible for the repression of CKs signaling in this region (Muller and Sheen, 2008). It might be possible that a similar crosstalk occurs in the tip region of the scutellum of maize: the high auxin response at the abaxial tip region may repress cytokinin activity (Chen et al., 2014).

In a more recent study the functional characterization of seven *ZmHistidine kinase* (*ZmHK*) genes, encoding the cytokinin receptors, was performed (Wang et al., 2014). Arabidopsis transgenic lines expressing each of the *ZmHK* genes showed a reduction in seed size with respect to the normal. This result suggests that *ZmHK*s function as repressors of seed development.

#### The role of brassinosteroids

Brassinosteroids (BRs) are plant steroid hormones involved in several developmental programs, including seed development. They function in the pathway that regulates ovule number and seed size and shape, in some cases complementing CKs and auxins. They also participate in the regulation of seed germination, by antagonizing the inhibitor effect of ABA (Zhang et al., 2009b), and being synergic to gibberellins (Leubner-Metzger, 2001).

The mechanisms by which BRs control seed development are still elusive. Many studies on BRs signaling have been performed on BR-deficient mutants in rice (Hong et al., 2005; Tanabe et al., 2005; Morinaka et al., 2006; Jiang and Lin, 2013). Different works demonstrated the relevance of BRs on seed size determination also in Arabidopsis, using BR-deficient or insensitive mutants (Choe et al., 2000; Li et al., 2001; Jiang et al., 2013; Jiang and Lin, 2013). Arabidopsis mutants deficient in BRs (i.e., *dwarf5, dwf5*; *dwf11*; *shrink1-D, shk1-D*; *detiolated2, det2*; *BR-Insensitive1, bri1*) produce dwarf phenotypes and smaller and fewer seeds (Chory et al., 1991; Choe et al., 2000; Takahashi et al., 2005; Jiang and Lin, 2013).

The control of seed development by BRs is mainly exerted through the function of the protein BRASSINAZOLE RESISTANT1 (BZR1). The activity of BZR1 varies according to its state of phosphorylation, dependent on the presence of BRs and mediated by the BIN2 kinase, which acts as a negative regulator (Li and Nam, 2002; Wang et al., 2002; Yin et al., 2002). BZR1 acts as a master regulator, from which a network of links extends to proteins acting in the different pathways of seed development and determination: ARF2, TRANSPARENT TESTA GLABRA2 (TTG2) and TTG16 regulating integument development; SHB1, IKU1, and 2, MINI3 together with AP2 and the MADS-box AGL61, 62, and 80 regulating endosperm development; and epigenetic regulators of endosperm development and paternal imprinting such as FIS2, MEA, FIE, MET1, SWN, and MSI1 (Sun et al., 2010; Jiang et al., 2013; Jiang and Lin, 2013). Indeed, the model proposed by Jiang and Lin (2013) indicates that BRs control the development of embryo and endosperm (and subsequently seed development) by regulating the expression of these genes through the direct or indirect action of BZR1.

The altered seed phenotype that *dwf5* and *shk1-D* mutants display indicates that BRs are also involved in seed shape determination (Choe et al., 2000; Takahashi et al., 2005). Thus, it has been observed that seed elongation requires BRs production and signaling in the maternal tissues (integuments) after fertilization. This aspect was especially evident in the *det2* mutant, where the cell length of the integuments was significantly reduced and partially rescued by exogenous application of BRs (Jiang et al., 2013).

Summarizing, in Arabidopsis it has been shown that the production of BRs in the embryo/endosperm is sufficient to increase seed volume, while they regulate seed size by an independent mechanism that involves BR production and signaling in the seed coat. How these hormones trigger different, localized behaviors in the different seed compartments still remains unknown (reviewed in Jiang and Lin, 2013).

Concerning maize, the information about BRs is extremely limited, and nothing is known about the conservation of the mechanism of action with respect to Arabidopsis and rice (Salas Fernandez et al., 2009). Hartwig et al. published one of the pioneering works in 2011. They characterized the mutant *nana plant 1 (na1)*, which is a mutant on *ZmDET2*, the Arabidopsis ortholog of *DET2* (Hartig and Beck, 2006; Hartwig et al., 2011). The phenotype of this mutant possesses all the characteristics of a BR-deficiency mutant, and shows the typical dwarfism. This work is complemented by a few other studies in which three additional genes involved in BRs biosynthesis and signaling were isolated (Tao et al., 2004; Liu et al., 2007b; Makarevitch et al., 2012). However, no data are available about the specific effect of BRs on maize seed development, so this is an attractive field to be explored.

#### The role of abscissic acid and gibberellins

The action of Abscissic acid and Gibberellins (GAs) on seed development is strictly correlated and antagonistic. As already mentioned, during the phase of seed maturation it is possible to define two important processes: the accumulation of nutrients in the endosperm (that will be used by the embryo during development and early phase of germination) and desiccation (that allow the embryo to tolerate hydric stress and terminate in the state of seed dormancy). Both processes are predominantly regulated by ABA. The concentration of this hormone increases during the late phase of seed maturation and is maintained until germination. In order to germinate, however, the seed must recover the water lost during maturation, since it is necessary to mobilize the resources for the embryo and activate enzymes and pathways for breaking of dormancy. This process, named imbibition, is regulated by the gibberellic acid. Hence, the ratio of ABA and GAs is determinant in the progression of seed maturation (Weber et al., 2005; Bethke et al., 2006; Seo et al., 2006; Liu et al., 2010).

The mechanism by which ABA controls the accumulation of food resources in the aleurone cell layer is based on the regulation of β –ZIP and DOF transcription factors (Vicente-Carbajosa and Carbonero, 2005; Monke et al., 2012). A study conducted by Karssen et al. (1983) and subsequently confirmed by Kanno et al. (2010) revealed that the biosynthesis of ABA occurs both in the maternal tissue and in the zygote. The synthesis of the hormone in the first compartment determines a primary peak of ABA accumulation, which is necessary to complete the process of embryo development. The final stages of embryo and endosperm formation correlate with a second peak of ABA, necessary to begin the process of seed desiccation and food storage in the aleurone layer (Kanno et al., 2010). Regarding other aspects of seed development, it is still unknown if ABA has additional functions in the zygotic compartments. In Arabidopsis, similarly to maize, it has been shown that the synthesis of starch and thus its accumulation begins at the early stage of seed development (Chen et al., 2011). During this process the synthesis of starch is regulated by phytohormones, such as ABA and IAA, with a major role played by ABA. It has been reported that the genes for starch metabolism (i.e., *AGPase, SS, DBE*, and *SBE*) are regulated by sugars (Rook et al., 2001; Bossi et al., 2009; Seiler et al., 2011). Moreover, interesting results by Hu et al. (2012) showed a correlation between *ZmSSI* (a sugar-regulated gene for starch-synthesis) and ABA during the endosperm filling stage. Identification of a sequence in the promoter of *ZmSSI* that is recognized by ABSCISIC ACID INSENSITIVE 4 (ABI4) allowed the characterization of the mechanism by which ABA regulates the expression of the gene. ABI4 would act as a transcriptional activator of *ZmSSI* in response to ABA treatment.

GAs, conversely to ABA, promote germination by mobilizing the resources necessary for embryo development (Koornneef et al., 1982). This is supported by the fact that after the cotyledon stage, when the events of PCD in the endosperm begin to leave space to the expanding embryo, some of the genes related with GA-biosynthesis are activated, followed by the activation of proteolytic enzymes and α-amylases (Sreenivasulu and Wobus, 2013). The study of the synthesis of bioactive GAs during seed development revealed that the peak of GAs occurs just before that of ABA (Singh et al., 2010; Nadeau et al., 2011). High auxin concentration also triggers the production of bioactive gibberellin (Dorcey et al., 2009), (Figure 1).

## Epigenetic control of endosperm development by genomic imprinting

Although the epigenetic mechanism of regulation is beyond the scope of this review, it is worth mentioning it, since it is still linked to hormones accumulation and coordination. In this context, a prominent role is exerted by the epigenetic mechanisms that determine the parent-of-origin specific gene expression, described as *genomic imprinting*. The regulation of gene expression by imprinting has been extensively studied in maize and rice, as Monocots, and in Arabidopsis, representing Eudicots (Hsieh et al., 2009; Luo et al., 2011; Waters et al., 2011).

The process by which a gene is imprinted involves the placement of epigenetic marks on its genomic sequence (i.e., DNA methylation) or in the nucleosomes (i.e., histone modifications). Different classes of methyltransferases can deposit the DNA-methylation marks, however, the methyltransferase operating in the endosperm is mainly the cytosine-DNA-methyltransferase MET1 (Law and Jacobsen, 2010). The action of DEMETER (DME) is antagonistic to MET1 as it removes the methylation marks from specific imprinted genes in maternal tissues allowing their expression (Choi et al., 2002; Gehring et al., 2006). Histone H3K27 tri-methylation is the epigenetic mark for silencing. The proteins that catalyze this modification belong to the Polycomb group of proteins (PcG). Genes like *MEA/FIS1, FIS2*, and *FIE*/*FIS3* belong to the PcG, and exert a pivotal role on maintaining the pattern of gene expression in the endosperm. These proteins participate in the formation of the Polycomb Repressive Complex 2 (PRC2), contributing to the establishment of an imprinted state, thus controlling gene expression in the developing seed (Hennig and Derkacheva, 2009).

The process of imprinting takes place already during gametophyte formation (Reik and Walter, 2001; Feil and Berger, 2007; Waters et al., 2013b). Epigenetic mechanisms involving small interfering RNAs (siRNAs), which are responsible for silencing of the complementary gene targets, have been suggested as having a prominent role in genome-wide hypomethylation of Arabidopsis endosperm (Hsieh et al., 2009, 2011; Bauer and Fischer, 2011). In particular, it has been shown that genomic demethylation by DME induces the production of siRNAs, in both Arabidopsis (Ibarra et al., 2012) and rice (Rodrigues et al., 2013). It has been observed that an extensive process of demethylation on Transposon Elements (TE) or repeats participates in the process of imprinting by affecting the neighboring genes (Choi et al., 2002; Gehring et al., 2006). Indeed, in Arabidopsis, one third of the imprinted genes are flanked by TEs (Bauer and Fischer, 2011; Gehring et al., 2011; Wolff et al., 2011). The global hypomethylation in the endosperm is likely originated from the central cell nucleus of the female gametophyte prior to fertilization (Ibarra et al., 2012). The major role of demethylation in female gametophyte (central cell) and male gametophyte (vegetative nucleus) is to produce siRNAs that are transported to the egg cell (in the female gametophyte) and the sperm cells (in the pollen) to reinforce the silencing of TEs (Gutierrez-Marcos et al., 2006; Bauer and Fischer, 2011; Ibarra et al., 2012). Given that, prior to fertilization the parental alleles are differentially methylated in each gametophyte, the triploid endosperm generated in the second event of fertilization will cope with a different state of methylation between the parental alleles. The different state of methylation between the alleles is believed to trigger the establishment of genomic imprinting in specific parental genes (Raissig et al., 2011). Interestingly, the control of gene expression by imprinting is important in the context of the determination of seed size and viability of the embryo (Xiao et al., 2003).

Despite that few imprinted genes are common between maize, rice and Arabidopsis, the mechanism that regulates parent-of-origin expression seems to be conserved between Monocots and Eudicots (Gutierrez-Marcos et al., 2006; Rodrigues et al., 2013). It is well established that the major site where the imprinted genes accumulate is the endosperm (Luo et al., 2011). Nevertheless, in both Arabidopsis and maize imprinted genes have also been identified in the embryo (i.e., *maternally expressed in embryo1, mee1* Jahnke and Scholten, 2009). The identity of the imprinted genes in both Arabidopsis and maize has been determined, in many cases, through advanced techniques of transcriptome deep sequencing and genome-wide search (Gehring et al., 2011; Waters et al., 2011; Zhang et al., 2011, 2014; Xin et al., 2013). These genes encoded proteins with a wide range of molecular functions, ranging from the regulation of pigmentation, starch metabolic pathways, protein storage, hormone responses, cell wall formation, transcriptional regulation, chromatin modification, and cytoskeletal function to mRNA regulation.

The distinct pattern of Maternally Expressed Genes (MEGs) and Paternally Expressed Genes (PEGs) ensures the proper evolution of seed development and is associated with specific stages of endosperm development (Raissig et al., 2013). In maize, the imprinted gene *Meg1* is involved in nutrient allocation and has a role in controlling seed size (Costa et al., 2012). *Meg1* positively regulates transfer cell specification and development, therefore increasing nutrient uptake. This effect is especially pronounced at 10 DAP, when a peak of auxin in BETL, ESR and aleurone layer coincides with the onset of nutrient accumulation for starch and zein storage protein synthesis (Lur and Setter, 1993a; Sabelli and Larkins, 2009; Forestan et al., 2010). The involvement of imprinted genes in hormone signaling pathways and transcriptional regulation of endosperm development is evidence of the importance of auxin in correct seed development (Xin et al., 2013). The role of imprinted genes (in particular MEGs) in driving the correct nutrient synthesis is coupled with the importance of the transport mediated by auxin. It is not surprising that *PIN1* was identified as specific MEG (Xin et al., 2013). Interestingly, *YUC10* orthologs in rice, maize and Arabidopsis were found to be exclusively paternally expressed, meaning that the function of YUC10 could be crucial for endosperm development (Waters et al., 2013b). The relevance of MEGs and PEGs in this stage of development is to facilitate the communication and coordination between endosperm, embryo and maternal tissues.

### miRNAs control of seed development

Another relevant mechanism controlling gene expression during seed development is exerted by microRNAs (miRNAs). miRNAs are single-stranded RNA molecules of 21–22 nucleotides in length (with some exceptions) processed from a precursor molecule defined as pre-miRNA (Bartel, 2004). The RNA duplex is subsequently transported out of the nucleus, where the complementary sequence (called star) is removed to allow mature miRNA to be ready for action. The mature miRNA binds with perfect or imperfect complementarity to sites in the 5′ or 3′ untranslated regions (UTR) or coding sequences (CDS) of genes, causing its cleavage or translational repression (Grennan, 2008).

Plant microRNAs play important regulatory roles in many biological and metabolic processes, including development, hormone signaling, and responses to environmental stress (Reinhart et al., 2002). miRNAs are frequently grouped in families that have a specific set of transcript targets and appear to be evolutionarily conserved between plant species (Wu et al., 2009; Wang et al., 2011). It has been observed that miRNAs are expressed from early to later stages during seed development (Kang et al., 2009; Nodine and Bartel, 2010; Rubio-Somoza and Weigel, 2011). More specifically, they seem to be implicated in the control of embryogenesis and embryo patterning, also affecting the germination process (Seefried et al., 2014). Mutants lacking essential components of miRNA biogenesis and/or processing, manifest a severely compromised seed development or even lethality (Nodine and Bartel, 2010). The relevance of miRNAs function in the control of seed development is especially evident by observing *dicer-like1* mutants, in which alterations are mainly at the level of embryo apical-basal-radial symmetry (Lang et al., 1994; Ray et al., 1996; Schauer et al., 2002).

During the process of seed maturity miRNAs also affect seed size. During gametophyte and early seed development, for example, miR172 targets several *APETALA2*-like transcription factors, thus controlling seed size and yield; *ap2* loss-of-function causes an increase of seed weight (Jofuku et al., 2005; Tang et al., 2012). The miR172-*AP2* interaction is conserved between Arabidopsis and maize (Wang et al., 2005).

Others miRNAs affecting seed size belong to two families, miR159 and miR319 (Palatnik et al., 2007; Li et al., 2011). *miR159ab* double mutant manifests a reduced seed size and seed shape alterations (Allen et al., 2007). miR319 has a central role in coordinating multiple miRNAs and is strictly connected to phytohormone regulation (Luo et al., 2011). This last seems to be an upstream regulator that targets several TCP transcription factors, which in turn activate the hormonal machinery through the recruitment of other miRNAs (Palatnik et al., 2003; Luo et al., 2011). The function of miR319 seems to be conserved also in maize where, in addition, this specific miRNA together with miR171 target genes that participate in secondary pathways of auxin and GA signaling transduction, thus affecting embryo differentiation (Zhang et al., 2009a; Kang et al., 2012; Shen et al., 2013).

Regarding the control exerted by miRNAs on phytohormones, it has been shown in Arabidopsis that auxin metabolism is controlled by at least four conserved miRNA families (miR160, miR167, miR390, and miR393), which mainly exert control by regulating ARF proteins (i.e., ARF6, ARF8, ARF10, ARF16, and 17) (Rhoades et al., 2002; Mallory et al., 2005; Marin et al., 2010; Windels and Vazquez, 2011; Kinoshita et al., 2012).

It has been observed that miRNAs in some cases also participate in the hormones cross-talk. For instance, it was reported that auxin and ABA signal transduction pathways are targets of differentially expressed miRNAs (Reyes and Chua, 2007). It was shown that miR159 and 160 affect the process of germination by regulating ABA sensitivity. Interestingly, miR160 is implicated in the regulation of auxin metabolism (Rhoades et al., 2002; Mallory et al., 2005); the mutants expressing a miR160-resistant form of ARF10 are hypersensitive to ABA (Liu et al., 2007a), therefore suggesting a point of cross-talk between ABA and auxin in imbibed seeds.

A deep sequencing approach has been used to identify seed specific miRNAs in maize (Zhang et al., 2006, 2009a; Wang et al., 2011; Kang et al., 2012). Most miR167 and miR319 families were found enriched in seeds rather than leaves (Kang et al., 2012). Target prediction of maize miRNAs found that miR167, as in Arabidopsis, targets ARFs (Zhang et al., 2009a). The conservation of both targets and miRNA in Arabidopsis and maize suggests conserved mechanisms of regulation in Monocots and Eudicots.

Another important example of conservation was recently reported in a study of miRNA regulation during the early development of barley grains. In this case, it was suggested that the miRNAs contribute to the control of the development of cereal grain particularly regulating phytohormone response pathways (Curaba et al., 2012). Specifically, the regulation of *TIR1* and potentially three *ARFs* by the miR393 and miR167 families resulted conserved.

The more recent knowledge on miRNAs and their molecular connections and involvement in multiple hormonal responses and crosstalk, with patterning genes in specific developmental processes and also in seed development is discussed in detail in other excellent reviews (Nonogaki, 2010; Rubio-Somoza and Weigel, 2011; Curaba et al., 2014).

## Concluding remarks

Spermatophytes have evolved seeds to ensure spreading and survival. Seed development and the inherent establishment of the final seed size is a multi-step process controlled by a complex network. Besides being a valuable model for basic research, it is also an important and interesting trait for farmers and the related industries, as seeds represent the basis for the food, feed and bio-based economy. However, despite the enormous advance on the study of the mechanisms controlling seed size, there are still open questions to answer. The general interest of the industry nowadays, apart from increasing production, is to modify the compounds accumulated in the seeds in order to improve their quality. With this perspective in mind, in these last years, many researchers have focused their attention on comparative studies between species, and the translation of information from model plants to crops. In this review we have summarized the known genes and elements involved in the process of seed development, particularly focusing on the auxin hormonal pathway, and underlining the concept that the proper distribution, transduction and coordination of all the hormonal signals is essential for seed development.

The early stages of the seed developmental program are very similar in Eudicots and Monocots. Nevertheless, remarkable differences have been described in the stage of seed maturation: while in Monocots the endosperm persists until maturation, being the compartment of nutrient storage for the embryo, in Eudicots it is gradually replaced by the embryo until being reduced to a single cell layer (Figure 1).

The initial genetic studies on seed biology aimed the identification and characterization of genes involved in fundamental processes associated with embryogenesis and seed development, based mainly on the characterization of mutants, misexpression experiments and the use of marker lines. Most of the genes identified showed relationship with phytohormones (Table 1). The knowledge of the pathways and events regulated by each hormone leads to the interesting possibility of specific hormones/hormone inhibitors being applied to the crops at specific moments in their development, in order to obtain the desired results in agricultural production.

Phytohormones are among the most relevant signals involved in the communication between seed structures. The communication among the three compartments of the seed (seed coat, endosperm and embryo) has been revealed to have a key role for the correct seed formation. In fact, failure in this communication can cause alteration in seed size, defective embryos and future seedlings and, in extreme cases, seed abortion. The characterization of mutants affecting hormonal signaling constitutes an interesting opportunity to describe relationships, relevance and mechanisms by which the signals are exchanged between seed compartments.

The difficulty in studying the role of genes whose mutations cause lethal phenotype in embryo/seed has recently been overcome with modern molecular biology techniques and by the use of *-omics* approaches. As mentioned in this review Lu et al. (2013), through the massive study of gene expression by RNA-sequencing, contributed to the identification of genes differentially expressed between embryo and endosperm, proposing interesting regulatory networks between the two compartments, in maize.

Several comparative studies between species are allowing the identification of the conserved and divergent elements acting in the regulatory mechanisms governing seed development. As shown in this review most of the crosstalk involving the factors that affect seed development (hormones, epigenetics and sugars) are conserved between maize and Arabidopsis (Figure 3). In addition, the identification and characterization of orthologous genes will permit the description of unknown genes function, and the definition of patterns of regulation that would otherwise be difficult to describe. For instance, the recent study by Chen et al. (2014) highlighted the different patterns of auxin and cytokinin accumulation during embryogenesis in the two model species Arabidopsis and maize. While in Arabidopsis the involvement of IAA in embryo development immediately after fertilization is clear, in maize this hormone is not detected in the embryo, but in the endosperm, during early embryogenesis. This result indicates the presence of a *phase shift* in the level of IAA accumulation during the early stages of embryo development when comparing the two species. Nevertheless, later in the development, the same pattern of hormone accumulation is observed at both scutellum (maize) and at the emerging cotyledon tips (Arabidopsis). The polar transport and the consequent IAA flux, which is essential in establishing the apical-basis pattern of the embryo, are conserved in both species as well. However, in maize after the differentiation of embryo meristems, auxin polar transport is also essential for the regular differentiation of both the leaf primordia at the SAM and the seminal root at the root apical meristem.

Similarly to auxin, cytokinin response is delayed in maize with respect to Arabidopsis. However, the antagonistic effect of auxin and cytokinins during embryogenesis is again conserved in these two species. The existence of a functional convergence in seed development between Arabidopsis and maize was moreover deduced by observing the phenotype of the Arabidopsis triple receptor kinase mutant *ahk2/ahk3/ahk4* and the effect of *ZmHK*s overexpression in Arabidopsis transgenic lines. The seeds showed an increased size with respect to the wild type in the triple mutant while a reduction in size in the overexpressing lines.

At the later stages of seed development, ABA and IAA seem to co-regulate the expression of many genes involved in starch biosynthesis in maize endosperm and the accumulation of nourishing proteins in the arabidopsis cotyledons. This evidence underlines the importance of auxin during all the stages of seed development, from embryogenesis to maturation (Figure 1).

Although some of the factors controlling the seed development process have been identified, many are still unknown. For example, to date, there is no information available about the role of brassinosteroids in maize seed development.

The arduousness of these studies in maize is not only linked to methodology-limitation, but also mainly attributable to genomic complexities. In cereal crops, the high level of gene duplication with respect to Arabidopsis hinders the identification of orthologs, or the simple comparison of gene function. In Monocots several cases of sub-functionalization were reported. In maize, for example, it was shown that the *PIN* gene has been subjected to events of duplication that generated the four *ZmPIN1a-d* genes with different levels of tissue-specific expression. It has been suggested that these four genes could have gained new functions assembling the specific roles of *AtPIN3, AtPIN4*, and *AtPIN7*, which have still not been identified in the maize genome. Despite the problems concerning the study of crops with respect to the Eudicot model plants, specific areas of research are improving in Monocots. One of these is the characterization of the process of genome imprinting. This study is more advanced in maize than in Arabidopsis, due to the bigger seed size, the persistence of the endosperm and the straightforward physical separation of the seed structures.

Imprinting is fundamental for both embryo and endosperm differentiation during seed development, which interestingly is the sole plant developmental phase characterized by maternal dependency. The study of the dynamic of the imprinted genes during early and late stages of development, and their cross-talk with hormones will shed light on both the biological significance of this mechanism of gene expression regulation, and the connection between epigenetic mechanisms and hormonal control during seed development and maturation. The identification of miRNAs differentially expressed in the seed and their corresponding targets has established a new complicated link between miRNAs dynamics and the traditional role of hormones in seed development. However, the study of conservation of both miRNAs and target gene expression between different species during seed development still needs further investigations.

The use of the present and new marker lines and mutants, as well as the technical advances on transcriptomics, proteomics, genomics and metabolomics, will greatly contribute to the understanding of the environmental, hormonal, genetic, and epigenetic mechanisms and cross-talks that control seed production and development, and to establish parallelisms between Monocots and Eudicots in the near future.

### Conflict of interest statement

The authors declare that the research was conducted in the absence of any commercial or financial relationships that could be construed as a potential conflict of interest.
